# Screening for Diabetic Retinopathy and Nephropathy in Patients with Diabetes: A Nationwide Survey in Korea

**DOI:** 10.1371/journal.pone.0062991

**Published:** 2013-05-08

**Authors:** Sang-Ho Byun, Seung Hyun Ma, Jae Kwan Jun, Kyu-Won Jung, Boyoung Park

**Affiliations:** 1 Department of Preventive Medicine, Seoul National University College of Medicine, Seoul, Korea; 2 National Cancer Control Institute, National Cancer Center, Goyang-si, Gyeonggi-do, Korea; Universidad Peruana Cayetano Heredia, Peru

## Abstract

This study was performed to identify factors associated with screening for diabetic retinopathy and nephropathy. Data from the Korean National Health and Nutrition Examination Survey between 2007 and 2009 were analyzed. Of 24,871 participants, 1,288 patients diagnosed with diabetes at ≥30 years of age were included. 36.3% received screening for diabetic retinopathy, and 40.5% received screening for diabetic nephropathy during the previous year. Patients living in rural areas, those with less education, those who had not received education about diabetes care, and those who did not receive medical care for diabetes were screened less often for retinopathy or nephropathy. Patients with poorer self-reported health status were screened more often. Occupation, smoking status, and diabetes duration were associated with retinopathy screening. Lower family income was associated with decreased nephropathy screening. Receiving education about diabetes care and receiving medical care for diabetes were significant factors in patients with a shorter duration of diabetes (the significant odds ratio [OR] of not receiving education varied between 0.27 and 0.51, and that of not receiving medical care varied between 0.34 and 0.42). Sociodemographic factors and health-related factors as well as education and medical care influenced screening for diabetic complications among those with a longer duration of diabetes (for retinopathy and nephropathy, the significant OR of living in a rural area varied between 0.56 and 0.61; for retinopathy, the significant OR of current smokers was 0.55, and the *p*-trend of subjective health status was <0.001; for nephropathy, the significant OR of a monthly household income of <3000 dollars was 0.61 and the *p*-trends of education and subjective health status were 0.030 and 0.007, respectively). Efforts to decrease sociodemographic disparities should be combined with education about diabetes care to increase the screening, especially for those with a longer duration of diabetes.

## Introduction

Diabetes is one of the most common and rapidly increasing chronic diseases globally, and has been called an “diabetes epidemic”. The prevalence of diabetes was estimated to be 2.8% in 2000 and is expected to increase to 4.4% by 2030, indicating that the number of the people living with diabetes will increase from 171 million in 2000 to 366 million in 2030 [1]. The increased prevalence of diabetes is attributable to an aging population, changes in lifestyle with economic development, and increasing rates of obesity [2]. In Korea, the prevalence of diabetes rose from 7.6% in 2001 to 9.1% in 2005, and that of impaired fasting glucose was 17.4% in 2005 [3]. Additionally, the economic burden of diabetes in Korea is increasing, as annual direct costs were 260 million dollars in 2009 [4].

Diabetes is always associated with long-term complications. Preventing complications is important because of the morbidity, mortality, and health care costs associated with diabetes complications [2], [5], [6]. Microvascular complications such as diabetic retinopathy and diabetic nephropathy are common. The prevalence of diabetic retinopathy among patients with diabetes is 34.6% worldwide [7]. Diabetic nephropathy develops in 40% of type 2 diabetic patients and characterized by persistent albuminuria [8] and diabetic nephropathy is the single most common cause of end-stage renal disease [9]. Progression to microalbuminuria occurs at 2.0% per year, and about 25% of patients with diabetes develop microalbuminuria or worse nephropathy within 10 years of diagnosis [10]. Although early retinopathy and microalbuminuria are associated with low health care costs, they can progress to more costly advanced diseases such as blindness and end-stage renal disease [6], [11].

An initial and annual dilated and comprehensive eye examination and urine albumin excretion test are recommended for all patients with type 2 diabetes [12]. Laser photocoagulation reduces blindness due to retinopathy, and more patients would benefit if the treatment were delivered at a sufficiently early stage. Interventions such as angiotensin-converting enzyme inhibitors and angiotensin II type I receptor blockers during the microalbuminuria stage diminish the risk of renal failure. Therefore, early detection of diabetic complications through regular screening is important for diabetes care. However, previous studies conducted in Korea have shown that only 39% of people with diabetes received a dilated eye examination, and only 51% underwent a microalbuminuria test during the previous year [13]. Considering the low screening rate for diabetic complications, it is important to identify high-risk groups that do not receive screening. This study was conducted to identify the factors that were associated with screening for diabetic retinopathy and nephropathy in the previous year through an exploratory study.

## Materials and Methods

### Data Source and Subjects

This study was based on data from the fourth Korean National Health and Nutrition Examination Survey IV (KNHANES IV) conducted between 2007 and 2009. The KNHANES is a nationally representative, cross-sectional survey to estimate the health and nutritional status of the Korean population. The target population is civilian non-institutionalized Koreans of at least 1 year of age. The study used a rolling sampling design with stratified multistage cluster probability sampling according to geographic area, age, and gender. The KNHANES IV consisted of a health interview, health examination survey, and nutrition survey. Details of the survey are described elsewhere [14], [15].

**Figure 1:**
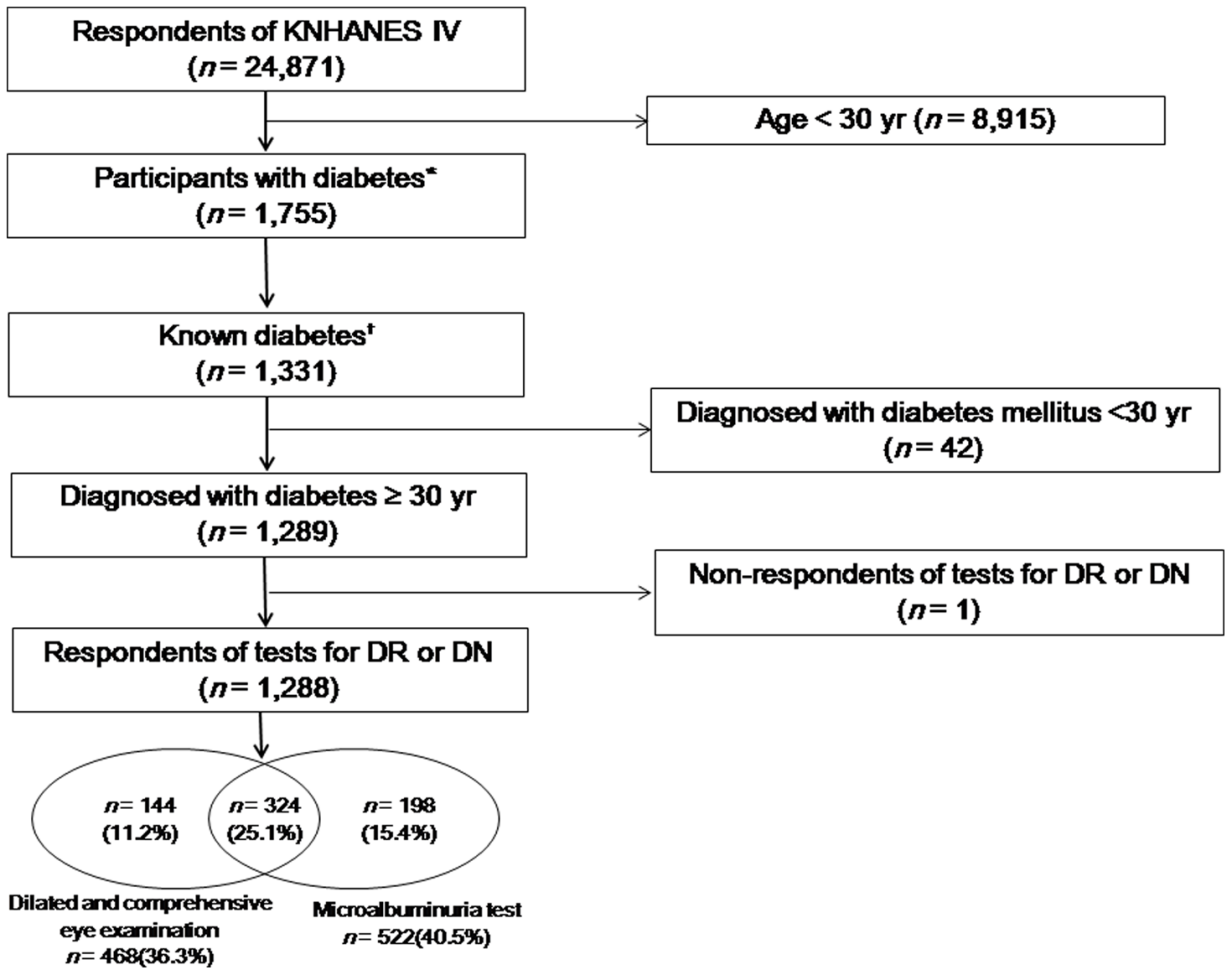
Study population framework. KNHANES, Korean National Health and Nutrition Examination Survey; DR, diabetic retinopathy; DN, diabetic nephropathy. *Subjects with fasting plasma glucose ≥126 mg/dL or a previous diagnosis of diabetes by a clinician, or who were taking insulin or oral antidiabetic medication. ^†^Subjects diagnosed with diabetes by a clinician or taking insulin or oral antidiabetic agents.

A total of 31,705 individuals were sampled for the KNHANES IV, and 24,871 participated in the survey, for a response rate of 78.4%. Individuals with diabetes were defined as those with fasting plasma glucose ≥126 mg/dL or with a previous diagnosis of diabetes by a clinician, or those taking insulin or oral antidiabetic medication. To restrict “known diabetes,” we excluded diabetic individuals who had never received a clinical diagnosis and were not taking insulin or oral antidiabetic agents [16]. Among “known diabetic” subjects, those who were diagnosed at ≥30 years of age were included in the analysis. Of the 24,871 participants in the health survey, we excluded those <30 years old, and 1,755 subjects who had diabetes and were ≥30 years old were identified. 1,331 of whom were “known diabetics.” We additionally excluded 42 subjects who were diagnosed with diabetes before the age of 30 years. One subject was excluded due to a non-response to the screening for both tests (Fig. 1). Finally, 1,288 respondents were included in this analysis. Ethics approval was not needed because the survey data are publicly available to download from the KNHANES site (http://knhanes.cdc.go.kr).

### Participant Data and Measurements

The participants were asked whether they had been diagnosed with diabetes by a clinician, the age at first diagnosis, treatment method, and whether they had received a dilated and comprehensive eye examination and microalbuminuria test during the previous year. Through face-to-face interviews by trained interviewers, sociodemographic information regarding sex, age, marital status, residential area, education, monthly household income, occupation, insurance type, and private insurance for health care, health-related factors such as self-rated health status, and health behavioral risk factors such as alcohol consumption and smoking were collected. Those who were prescribed insulin or oral antidiabetic agents were defined as receiving medical care for diabetes. Also, whether they had been educated about diabetes care by clinicians or the public health sector was asked. After a 12-hours overnight fast, blood samples were drawn, and the fasting plasma glucose level was measured. The glycated hemoglobin (HbA1c) level was measured by high performance liquid chromatography. Based on the HbA1c level, the controlled group comprised patients who maintained an HbA1c level of <6.5%. Those with an HbA1c level of ≥6.5% were assigned to the uncontrolled group.

### Statistical Analysis

The basic characteristics of the subjects with diabetes are presented as numbers and percentages. Factors independently associated with receiving diabetic retinopathy screening by dilated and comprehensive eye examination and diabetic nephropathy screening by microalbuminuria testing during the previous year were evaluated using multiple logistic regression. To avoid over-adjustment by including an excess number of variables, only those variables with a statistical significance level <0.1 in the simple logistic regression were included in the multiple logistic regression. For diabetic retinopathy screening, the multiple logistic regression included sex, age, residence area, education, occupation, self-reported health status, alcohol consumption, smoking, education about diabetes care, medical care of diabetes, diabetes control, and diabetes duration. For diabetic nephropathy screening, the multiple logistic regression included residence area, education, monthly family income, occupation, self-reported health status, smoking, education about diabetes care, medical care for diabetes, and diabetes duration. We conducted a subgroup analysis to identify factors associated with receiving screening for diabetic retinopathy and diabetic nephropathy by duration of diabetes. Although previous studies have applied various cut-offs [17]–[19], we used a duration of 5 years as a cut-off (≤5 years and >5 years) as a previous study conducted in Korea [18] because this value divided our data in half. In this analysis, only variables with a statistical significance <0.1 were included in the multiple logistic regression. For those whose duration of diabetes was ≤5 years, the multiple logistic regression for diabetic retinopathy screening included monthly family income, occupation, smoking, education about diabetes care, medical care of diabetes, and diabetes control, while the multiple logistic regression for diabetic nephropathy screening included residence area, education, monthly family income, education about diabetes care, and medical care of diabetes. For those whose duration of diabetes was >5 years, the multiple logistic regression of diabetic retinopathy screening included residence area, education, occupation, self-reported health status, alcohol drinking, smoking, education about diabetes care, and medical care of diabetes, while the multiple logistic regression for diabetic nephropathy screening included residence area, education, monthly family income, occupation, self-reported health status, education about diabetes care, and medical care of diabetes. All statistical analyses were performed using SAS software version 9.2 (SAS Institute, Cary, NC, USA).

## Results

Of the 24,871 KNHANES IV participants, 1,288 known diabetics who were diagnosed at ≥30 years of age were included in the analysis. Their sociodemographic factors and health behavioral risk factors are presented in Table 1. Among the 1,288 diabetics, 36.3% (*n = *468) received screening for diabetic retinopathy, 40.5% (*n = *522) received screening for diabetic nephropathy, and 25.1% (*n = *324) received both tests during the previous year (Fig. 1).

**Table 1:** Basic characteristics of the study participants.

Characteristic		N	(%)
Sex	Male	595	(46.2)
	Female	693	(53.8)
Age (years)	≤59	406	(31.5)
	60–69	493	(38.3)
	≥70	389	(30.2)
Marital status	Married	944	(73.3)
	Single, bereaved, divorced	338	(26.2)
	Missing	6	(0.5)
Residence area	Urban area	891	(69.2)
	Rural area	397	(30.8)
Education	≤Elementary school	713	(55.4)
	Middle school	197	(15.3)
	≥High school	370	(28.7)
	Missing	8	(0.6)
Monthly family income	<3000 dollars	962	(74.7)
	≥3000 dollars	279	(21.7)
	Missing	47	(3.7)
Occupation	Managerial and professional	91	(7.1)
	Service and sales	88	(6.8)
	Routine and manual	347	(26.9)
	Unemployed/Housewives	743	(57.7)
	Missing	19	(1.5)
Insurance type	National Health Insurance	1157	(89.8)
	Medical Aid Program	119	(9.2)
	Missing	12	(0.9)
Private insurance for health care	Yes	505	(39.2)
	No	756	(58.7)
	Missing	27	(2.1)
Self-reported health status	Good	270	(21.0)
	Moderate	337	(26.2)
	Poor	680	(52.8)
	Missing	1	(0.1)
Alcohol drinking	Current drinker	635	(49.3)
	Ex-drinker/nondrinker	649	(50.4)
	Missing	4	(0.3)
Smoking	Current smoker	1058	(82.1)
	Ex-smoker/nonsmoker	224	(17.4)
	Missing	6	(0.5)
Educated about diabetes care	Yes	246	(19.1)
	No	1010	(78.4)
	Missing	32	(2.5)
Medical care of diabetes	Yes	1039	(80.7)
	No	249	(19.3)
Diabetes control	Controlled*	302	(63.3)
	Uncontrolled†	816	(23.5)
	Missing	170	(13.2)
Diabetes duration	≤5 years	633	(49.2)
	>5 years	655	(50.9)

* Glycated hemoglobin (HbA1c) level <6.5%;

† HbA1c ≥6.5%.

Table 2 describes factors associated with receiving screening for diabetic retinopathy. Subjects with poorer self-reported health status and a diabetes duration >5 years were screened significantly more often for diabetic retinopathy (P-trend 0.001; odds ratio (OR), 1.74 [95% confidence interval (CI), 1.34–2.25]). People living in rural areas, current smokers, those who had not received an education about diabetes care, and those who had not received medical care for diabetes were screened significantly less often for diabetic retinopathy (OR 0.65 [0.49–0.87]; OR 0.62 [0.43–0.91]; OR 0.36 [0.27–0.50]; OR 0.35 [0.24–0.53], respectively). A lower level of education was associated with a lower tendency for diabetic retinopathy screening (p-trend 0.031). Additionally, occupations other than managerial or professional were associated with a lower rate of dilated and comprehensive eye examination (service/sales jobs OR 0.41 [0.21–0.82]; routine/manual jobs OR 0.43 [0.25–0.76]; unemployed/housewife OR 0.53 [0.31–0.92]). Although the distributions of current smokers, self-reported health status, and education about diabetes care differed according to education level, we did not find any interaction effects between current smokers, self-reported health status, education about diabetes care, and education level in terms of diabetic retinopathy or diabetic nephropathy screening.

**Table 2:** Factors associated with receiving a dilated and comprehensive eye examination for diabetic retinopathy screening and microalbuminuria test for diabetic nephropathy screening during the previous year.

Factor	Diabetic retinopathy screening				Diabetic nephropathy screening			
	Simple logistic regression		Multiple logistic regression * ^,^ **		Simple logistic regression		Multiple logistic regression* ^,^ ††	
	OR	95% CI	OR	95% CI	OR	95% CI	OR	95% CI
Sex								
Male	1		1		1			
Female	1.25	(0.99–1.57)	1.19	(0.88–1.62)	1.07	(0.86–1.34)		
Age (years)								
≤59	1		1		1			
60–69	1.26	(0.96–1.67)	1.19	(0.85–1.67)	0.93	(0.71–1.21)		
≥70	1.39	(1.04–1.86)	1.44	(0.98–2.11)	0.83	(0.62–1.10)		
* p*-trend			0.061					
Marital status								
Married	1				**1**			
Single, bereaved, divorced	1.12	(0.86–1.44)			1.13	(0.88–1.46)		
Residence area								
Urban area	1		1		1		1	
Rural area	0.63	(0.49–0.82)	**0.65**	**(0.49**–**0.87)**	0.64	(0.50–0.82)	**0.69**	**(0.52**–**0.90)**
Education								
≤Elementary school	0.79	(0.61–1.02)	**0.68**	**(0.48**–**0.97)**	0.58	(0.45–0.74)	**0.61**	**(0.45**–**0.84)**
Middle school	0.88	(0.62–1.26)	0.95	(0.63–1.43)	0.70	(0.49–0.99)	0.76	(0.51–1.13)
≥High school	1		**1**		1		**1**	
* p*-trend			**0.031**				**0.002**	
Monthly family income								
<3000 dollars	0.80	(0.61–1.05)			0.67	(0.51–0.87)	**0.73**	**(0.53**–**0.99)**
≥3000 dollars	1				1		**1**	
Occupation								
Managerial and professional	1		1		1		1	
Service and sales	0.54	(0.29–0.99)	**0.41**	**(0.21**–**0.82)**	0.89	(0.49–1.60)	1.04	(0.55–1.98)
Routine and manual	0.47	(0.29–0.75)	**0.43**	**(0.25**–**0.76)**	0.67	(0.42–1.07)	1.05	(0.61–1.80)
Unemployed/Housewives	0.87	(0.56–1.35)	**0.53**	**(0.31**–**0.92)**	0.81	(0.53–1.26)	0.97	(0.58–1.63)
Insurance type								
National Health Insurance	1				1			
Medical Aid Program	1.31	(0.89–1.92)			1.00	(0.68–1.46)		
Private insurance								
Yes	1				1			
No	1.09	(0.86–1.38)			0.87	(0.70–1.10)		
Self-reported health status								
Good	1		1		1		1	
Moderate	1.04	(0.74–1.47)	1.09	(0.75–1.59)	1.46	(1.05–2.04)	**1.56**	**(1.09**–**2.22)**
Poor	1.50	(1.11–2.03)	**1.65**	**(1.18**–**2.30)**	1.52	(1.13–2.05)	**1.82**	**(1.32**–**2.51)**
* p*-trend			**0.001**				**<0.001**	
Alcohol drinking								
Current drinker	0.76	(0.60–0.95)	0.86	(0.65–1.13)	1.00	(0.80–1.25)		
Ex-drinker/nondrinker	1			1	1			
Smoking								
Current smoker	0.54	(0.39–0.75)	**0.62**	**(0.43**–**0.91)**	0.76	(0.56–1.03)	0.76	(0.55–1.06)
Ex-smoker/nonsmoker	1		**1**		1		1	
Educated about diabetes care								
Yes	1		1		1		1	
No	0.31	(0.23–0.41)	**0.36**	**(0.27**–**0.50)**	0.37	(0.28–0.50)	**0.42**	**(0.31**–**0.57)**
Medical care of diabetes								
Yes	1		1		1		1	
No	0.27	(0.19–0.39)	**0.35**	**(0.24**–**0.53)**	0.35	(0.25–0.48)	**0.37**	**(0.26**–**0.52)**
Diabetes control								
Controlled†	1		1		1			
Uncontrolled□	1.59	(1.20–2.11)	1.30	(0.95–1.78)	1.26	(0.96–1.65)		
Diabetes duration								
≤5 years	1		**1**		1		1	
>5 years	2.27	(1.80–2.87)	**1.74**	**(1.34**–**2.25)**	1.36	(1.08–1.70)	1.12	(0.88–1.43)

* Only variables with p-value <0.1 in the simple logistic regression were included in the multiple logistic regression;

† Glycated hemoglobin (HbA1c) level <6.5%;

□ HbA1c ≥6.5%;

** Adjusted for sex, age, residence area, education, occupation, self-reported health status, alcohol drinking, smoking, education about diabetes care, medical care of diabetes, diabetes control, and diabetes duration;

†† Adjusted for residence area, education, monthly family income, occupation, self-reported health status, smoking, education about diabetes care, medical care for diabetes, and diabetes duration.

A lack of education about diabetic care and a lack of medical treatment were the most influential factors associated with a lower rate of screening for diabetic nephropathy (OR 0.42 [0.31–0.57]; OR 0.37 [0.26–0.52], respectively). Living in a rural area (OR 0.69 [0.52–0.90]), having a lower education level (*p*-trend 0.002), and having a monthly family income of <3000 dollars (OR 0.73 [0.53–0.99]) were also associated with a lower rate of screening for diabetic nephropathy, whereas a poor self-reported health status was associated with a higher rate (*p*-trend<0.001) (Table 2).

Factors were also analyzed according to diabetic duration (≤5 y and >5 y) (Table 3). Regardless of diabetes duration, a lack of education about diabetic care and a lack of medical treatment were associated with lower screening rates for diabetic retinopathy and nephropathy. In contrast, living rural area (OR 0.56 [0.39–0.83]) and being a smoker (OR 0.55 [0.33–0.89]) were associated with a decreased screening rate for diabetic retinopathy, and poor self-reported health status (*p*-trend <0.001) was associated with an increased rate only in participants who had diabetes for >5 years. Similarly, for diabetic nephropathy screening, living in a rural area (OR 0.61 [0.41–0.89]), having a lower education level (*p*-trend 0.03), and having a lower household income (OR 0.61 [0.39–0.96]) were related to a lower rate, and poor self-reported health status (*p*-trend, 0.007) was associated with a higher rate only in subjects who had diabetes for >5 years. Among those who had diabetes for ≤5 years, occupation was the only factor related to the screening rate for diabetic retinopathy, and no factors exhibited an association with the screening rate for diabetic nephropathy.

**Table 3:** Factors associated with receiving screening for both diabetic retinopathy and diabetic nephropathy according to diabetes duration.

Factor*	Screening for diabetic retinopathy				Screening for diabetic nephropathy			
	≤5 years**		>5 years††		≤5 years†		>5 years***	
	OR	95% CI	OR	95% CI	OR	95% CI	OR	95% CI
Residence area								
Urban area			**1**		1		**1**	
Rural area			**0.56**	**(0.39**–**0.83)**	0.80	(0.55–1.16)	**0.61**	**(0.41**–**0.89)**
Education								
≤Elementary school			0.71	(0.45–1.11)	0.70	(0.47–1.05)	**0.60**	**(0.39**–**0.94)**
Middle school			0.87	(0.50–1.51)	0.83	(0.48–1.44)	0.68	(0.40–1.18)
≥High school			1		1		1	
* p*-trend			0.12		0.09		**0.030**	
Monthly family income								
<3000 dollars	0.65	(0.41–1.01)			0.82	(0.54–1.25)	**0.61**	**(0.39**–**0.96)**
≥3000 dollars	1				1		**1**	
Occupation								
Managerial and professional	1		1				1	
Service and sales	**0.35**	**(0.14–0.88)**	0.71	(0.25–1.98)			0.81	(0.28–2.29)
Routine and manual	**0.48**	**(0.24–0.98)**	0.55	(0.24–1.30)			1.02	(0.43–2.45)
Unemployed/housewives	0.63	(0.32–1.24)	0.82	(0.37–1.84)			0.94	(0.41–2.16)
Self-reported health status								
Good			1				1	
Moderate			1.43	(0.86–2.38)			**1.72**	**(1.03**–**2.86)**
Poor			**2.22**	**(1.43**–**3.45)**			**1.91**	**(1.23**–**2.99)**
* p*-trend			**<0.001**				**0.007**	
Alcohol drinking								
Current drinker			0.72	(0.50–1.03)				
Ex-drinker/nondrinker			1					
Smoking								
Current smoker	0.62	(0.36–1.07)	**0.55**	**(0.33**–**0.89)**				
Ex-smoker/nonsmoker	1		**1**					
Educated about diabetes care								
Yes	1		1		1		1	
No	**0.27**	**(0.16**–**0.43)**	**0.43**	**(0.28**–**0.64)**	**0.51**	**(0.32**–**0.82)**	**0.35**	**(0.23**–**0.52)**
Medical care of diabetes								
Yes	1		1		1		1	
No	**0.34**	**(0.20**–**0.58)**	**0.36**	**(0.19**–**0.67)**	**0.42**	**(0.28**–**0.63)**	**0.28**	**(0.15**–**0.52)**
Diabetes control								
Controlled†	1							
Uncontrolled?	1.24	(0.80–1.91)						

* Only variables with p-value <0.1 in the simple logistic regression were included in the multiple logistic regression and the results are shown in the table;

† Glycated hemoglobin (HbA1c) level <6.5%;

? HbA1c ≥6.5%;

** Adjusted for monthly family income, occupation, smoking, education about diabetes care, medical care of diabetes, and diabetes control;

†† Adjusted for residence area, education, occupation, self-reported health status, alcohol drinking, smoking, education about diabetes care, and medical care of diabetes;

† Adjusted for residence area, education, monthly family income, education about diabetes care, and medical care of diabetes;

*** Adjusted for residence area, education, monthly family income, occupation, self-reported health status, education about diabetes care, and medical care of diabetes.

## Discussion

Considering the disease burden of diabetes and its related complications, regular screening for complications according to guidelines is necessary not only to decrease diabetes-related burdens and cost but also to increase the quality of life for patients with diabetes. Although the screening guidelines for diabetic retinopathy and nephropathy are well established, many patients with diabetes are not referred for screening. The results of our study indicate that only 36.3% and 40.5% of patients received screening for diabetic retinopathy and nephropathy, respectively, during the previous year, even though they knew that they had diabetes. Receiving both tests annually is important considering that both retinopathy and nephropathy are common complications; nevertheless, only 25.1% of our subjects underwent both tests during the previous year. The rate of screening for diabetic retinopathy was similar to that in previous study; however, the rate of screening for diabetic nephropathy was significantly lower than that in previous study conducted in 2005 in Korea, in which 39.0% (95% CI = 36.2%–41.8%) and 50.9% (95% CI = 47.9%–53.9%) of patients with diabetes underwent screening for retinopathy and nephropathy, respectively, during the previous year [13]. The screening rates are lower in Korea than in other countries, in which the rates of retinopathy screening according to guidelines are 47–80% [19]–[21] and the 1-year screening rates for nephropathy are >60% [22]. Although the prevalence of diabetes is increasing, care for complications associated with diabetes appears to be declining in Korea, which shows a reverse trend compared with other countries where retinopathy screening rates according to guidelines are increasing [19], [23].

In the present study, residence area, education, occupation, self-reported health status, smoking status, education about diabetes care, diabetes medical treatment, and diabetes duration were factors associated with the frequency of screening for diabetic retinopathy. Among these, residence area, education, self-reported health status, diabetes care education, and medical treatment were also related to screening rates for diabetic nephropathy. Additionally, household income was associated with screening rates for diabetic nephropathy.

A previous study suggested that socioeconomic disparities such as those for education and income are associated only with retinopathy screening and not with nephropathy screening [24]. However, we found that socioeconomic disparities were significant in screening for both retinopathy and nephropathy. These results are consistent with earlier studies identifying a relationship between education and health behavior in the self-management of chronic diseases, and income could account for the education gradient [25]. A previous study regarding diabetes care also showed that lower education is associated with lower screening rates for retinopathy and nephropathy, but income showed no similar association [13]. Occupation may reflect socioeconomic status, and we found that those who did not have managerial or professional occupations were screened less often for retinopathy. In Korea, >80% of hospitals and primary care clinics are located in urban areas, and this inequality in the distribution of healthcare might have caused differences in screening rates for diabetic complications between urban and rural areas in this study. Thus, although National Health Insurance, which is a universal health insurance system covering nearly 100% of the Korean population, has been credited with lowering financial barriers to medical care [26], many socioeconomic barriers regarding screening for diabetic complications remain.

Many previous studies suggested that a lack of or inadequate knowledge regarding the necessity for retinopathy screening is the main barrier to receiving screening [27], and receiving diabetes education is associated with an increased screening rate for diabetic retinopathy [19], [20]. Disease management strategies are associated with better diabetes care such as higher retinal and nephropathy screening rates [28]. Our study showed that only 19% of known diabetic patients received education about diabetes care, and this was reflected in screening rates for diabetic retinopathy and nephropathy that were lower by 60–70%. The screening rate for diabetic retinopathy is about 72% in the U.S., where about 50% of patients have never been educated about diabetes care [29]. Thus, education programs about diabetes care that are presented by the public health sector or primary care centers should be strengthened. In previous studies, patients with a longer duration of diabetes [17], [27] and who received medical care [17], [20], [27] were more likely to be screened for retinopathy and nephropathy.

Visual problems and comorbidities are associated with receiving screening for retinopathy [19], [30], and self-rated health status, which was a significant factor in the present study, may be the aggregate of health problems, including visual problems and kidney problems. The goal of screening for diabetic retinopathy and nephropathy is to lower the rate of disease progression by early detection and intervention. Thus, to prevent development and aggravation of complications, patients with diabetes should receive screening according to recommendations. Smoking is related to the development of microvascular complications, including diabetic retinopathy and nephropathy, in patients with diabetes [31]. Our finding that smokers received diabetic retinopathy screening less often may be a problem for primary and secondary prevention of diabetic complications.

When we analyzed the subjects according to diabetes duration, education about diabetes care and medical treatment of diabetes were significant factors among those with a shorter duration of diabetes (≤5 y), and other sociodemographic factors did not influence screening, except for an effect of occupation on diabetic retinopathy screening. However, among those with a longer duration of diabetes (>5 y), not only diabetes care education and medical treatment but also residence area, education, income, self-reported health status, and smoking had effects on the screening rates for complications of diabetes. Thus, people with diabetes should be educated about diabetes care at the early diagnostic phase, and efforts to reduce sociodemographic disparities may be needed for those with a longer duration of diabetes.

This study had several limitations. First, the dependent variables were measured by self-report in health surveys, and we could not match the screening status with the medical record. Self-reported information regarding diabetic care is likely to overestimate the frequency of screening [32]. The accuracy of recall might have differed between diabetic retinopathy and nephropathy screening because the microalbuminuria test is usually performed in a primary care setting, whereas the dilated eye examination is performed mainly by ophthalmologists and requires a specific medical visit. Additionally, all of the independent variables except diabetes control (HbA1C) were self-reported. Therefore, we cannot rule out the possibility of information bias. Second, we assessed screening for diabetic retinopathy and nephropathy during the previous year; therefore, subjects who were screened regularly at intervals of >1 year were classified as not screened. Third, this study was a cross-sectional survey, and we cannot derive any conclusions on the causality of the associations.

Despite these limitations, this study has several strengths. First, because studies assessing factors associated with nephropathy screening in patients with diabetes are scarce, our results may contribute to increased understanding of diabetic care regarding diabetic nephropathy. Second, although previous studies focused on screening diabetic complications in patients, we also conducted a subgroup analysis according to diabetic duration, from which we suggest that efforts to increase the screening rate for diabetic complications should be distinguished according to disease duration. Furthermore, the results were based on patients with diabetes who were selected from a nationally representative general population.

## Conclusions

Although the prevalence of diabetes in Korea is increasing, diabetes care such as screening rates for diabetic retinopathy and nephropathy in Korea has not increased. We conducted an exploratory study to identify the factors that were associated with screening for diabetic complications. Socioeconomic and regional factors as well as other aspects of diabetic care such as education regarding diabetes care and medical treatment have influenced the uptake of screening for diabetic complications. Efforts to decrease sociodemographic disparities should be combined with education about diabetes care to increase the screening frequency for those with a longer duration of diabetes. Our results point to the need for a systematic approach to reducing socioeconomic, regional, and care-process disparities in the screening process and improving early diagnosis and management of diabetic complications in Korea.
